# Glutathione “Redox Homeostasis” and Its Relation to Cardiovascular Disease

**DOI:** 10.1155/2019/5028181

**Published:** 2019-05-09

**Authors:** Vladan P. Bajic, Christophe Van Neste, Milan Obradovic, Sonja Zafirovic, Djordje Radak, Vladimir B. Bajic, Magbubah Essack, Esma R. Isenovic

**Affiliations:** ^1^Laboratory for Radiobiology and Molecular Genetics, Institute of Nuclear Sciences Vinca, University of Belgrade, Mike Petrovica Alasa 12-14, 11000 Belgrade, Serbia; ^2^King Abdullah University of Science and Technology (KAUST), Computational Bioscience Research Center (CBRC), Thuwal 23955-6900, Saudi Arabia; ^3^Department of Vascular Surgery, Dedinje Cardiovascular Institute, Belgrade University School of Medicine, Belgrade, Serbia

## Abstract

More people die from cardiovascular diseases (CVD) than from any other cause. Cardiovascular complications are thought to arise from enhanced levels of free radicals causing impaired “redox homeostasis,” which represents the interplay between oxidative stress (OS) and reductive stress (RS). In this review, we compile several experimental research findings that show sustained shifts towards OS will alter the homeostatic redox mechanism to cause cardiovascular complications, as well as findings that show a prolonged antioxidant state or RS can similarly lead to such cardiovascular complications. This experimental evidence is specifically focused on the role of glutathione, the most abundant antioxidant in the heart, in a redox homeostatic mechanism that has been shifted towards OS or RS. This may lead to impairment of cellular signaling mechanisms and elevated pools of proteotoxicity associated with cardiac dysfunction.

## 1. Introduction

Glutathione (GSH) and its reduced form are the most prevalent thiol-containing peptides in eukaryotic cells [1, 2]. Although GSH was described as a prominent reducing factor and the main antioxidant within the cells, subsequent investigations show that GSH exerts many other cellular functions [2, 3]. Indeed, GSH exerts multiple physiological functions including the proliferation, cell cycle regulation, apoptosis, catabolism of xenobiotics, glutathionylation of proteins, and the production of some steroids, lipid compound, and deoxyribonucleotides and represents an important source of cysteine [2–5]. Taking into account all these features of GSH, it is not surprising that GSH plays an important etiological role in the development of numerous diseases, such as cardiometabolic and cardiovascular diseases (CVD) [6–9].

Development and progression of CVD are characterized by substantial changes in the concentration of GSH and/or its oxidation state [9–12]. Three different mechanisms have been proposed to be involved in GSH diminution: increased oxidation by intracellular oxidizing agents, increased conjugation to proteins, electrophiles, and xenobiotics, and increased extrusion across the cell membrane [9, 10, 13]. Conversely, increased concentration of GSH in cells may cause negative effects, such as multidrug resistance [9, 14]. Also, the dysregulation of GSH-dependent enzymes and GSH synthesis enzymes was observed in endothelial dysfunction [10, 12].

This review aims to highlight the role of GSH in the physiology and pathology of the cardiovascular system.

## 2. Glutathione Structure and Function

GSH is a peptide ubiquitously present in all cells, but the liver remains the principal source of GSH in humans [1, 2]. GSH is a tripeptide formed from glycine, glutamate, and cysteine. In the cell, GSH is synthesized and mostly distributed in the cytoplasm, while in less amount, it is also found in the organelles such as the nucleus, peroxisomes, mitochondria, and endoplasmic reticulum. GSH is transported from the cytoplasm to the organelles by specific transporters [2, 8, 15]. In many cells, the concentration of GSH is in the range of 1-10 mM, while the concentration of GSH in plasma is notably low at 0.01 mM [1]. This disproportionate level of GSH concentration within cells and circulation principally depends on its rapid catabolism in blood [8, 16]. However, it is not possible to determine the total glutathione concentration, which includes GSH, the glutathione disulfide (GSSG), and all other forms [17].

Various factors regulate GSH synthesis, such as availability of L-cysteine and ATP and the concentration of GSH [1, 2]. A large amount of GSH competitively inhibits the activity of glutamate cysteine ligase (GCL) [18, 19]. GSH is synthesized by GCL and glutathione synthetase (GS) [1, 2]. Firstly, *γ*-glutamylcysteine is formed from glutamate and cysteine via GCL activity and consumption of one ATP molecule. Then, glycine is added to formed dipeptide in the reaction catalyzed by GS and consumption of one more ATP molecule. Interestingly, the expression of GCL is regulated by nuclear factor erythroid 2-related factor 2 (Nrf2), which can be activated by oxidative stress (OS) [16, 20]. Thus, OS leads to increased GSH production through stimulation of GCL activity [16, 20]. One of the characteristic structural features of the GSH is the *γ*-glutamyl bond, which makes GSH very stable and resistant to cleavage of most proteases and peptidases. *γ*-Glutamyl transferase (GGT) is the enzyme which catabolizes GSH (located extracellularly) by removing *γ*-glutamyl to obtain cysteinylglycine or cysteinylglycine conjugates, which dipeptidases further degrade [17].

GSH is the principal intracellular antioxidant, which may act directly by scavenging reactive oxygen and nitrogen species or indirectly by supporting enzymatic activity as a cofactor [3, 21]. Intracellular GSH mainly exists as a monomer in reduced form and less in the disulfide dimer or GSSG, which arises after GSH oxidation [1, 3]. GSH can be reverted from GSSG by the activity of glutathione reductase [13, 22]. The reduced and oxidized forms of GSH represent the main cellular redox buffer, and in the physiological condition, the concentration of GSH is predominant compared with GSSG [23, 24]. Thus, the ratio of GSH and GSSG is considered as a marker of OS [23, 24].

Furthermore, glutathionylation of proteins represents an important regulatory mechanism that influences the activity and kinetics of different regulatory, metabolic, and structural proteins [25, 26]. Proteins with thiol groups can respond to different stimuli, such as OS, and form disulfides [26]. S-Thiolation processes include the formation of a disulfide bond inside of one protein and between two proteins and mixed protein/nonprotein disulfides. It was observed that GSH forms part of almost 85% of mixed protein/nonprotein disulfides [9, 26, 27]. We today view the process of S-glutathionylation as a critical signaling system in CVD [28]. S-Glutathionylation is involved in oxidative phosphorylation, myocyte contraction protein synthesis, and insulin response [29]. Perturbations in protein glutathionylation contribute to myocardial infarction, hypertrophy, and inflammation. Using the ischemia-perfusion technique in the rat model for myocardial infarction, it was shown that there is an increase in overall protein glutathionylation [30]. The protein found to be heavily glutathionylated was glyceraldehyde-3-phosphate dehydrogenase. The result of glutathionylation is inhibition of glycolysis and increased apoptosis [31]. Ras glutathionylation has been investigated in the progression of cardiac hypertrophy [32]. More research has been concerned with the role of protein glutathionylation in atherosclerosis [33–35]. Human macrophages exposed to oxidized cholesterol, a fundamental component of the atherosclerotic plaque, show an increase in protein glutathionylation [36] suggesting that protein glutathionylation has a role in macrophage cell death [36]. Patients with atherosclerosis obliterans or atherosclerosis of the extremities exhibit increased levels of serum proteins that have been seen to be heavily glutathionylated [37]. These findings reflect a redox imbalance produced by OS and present a path leading to atherosclerosis of the extremities.

Past research has been concentrated on OS and its relation to CVD [38], but new studies have given light to the role of reductants that may lead to the imbalance of normal, physiological production of reactive oxygen species (ROS) to a state of “reductive stress” (RS). S-Glutathionylation of proteins, in this new light, has to be included in the analysis of how to control OS and/or RS [38].

Regulating angiogenesis is a major goal in cardiovascular research. Research into S-glutathionylation on the regulation on the low molecular weight protein tyrosine phosphatase (LMW-PTP) which is a key mediator of vascular endothelial growth factor (VEGF) cell migration [39] was reported. VEGF causes reversible S-glutathionylation of the LMW-PTP protein. Research showed that a balanced redox state is needed for VEGF to process reversible S-glutathionylation of the LMW-PTP protein and hence cell migration. On the other hand, it was shown that a shift towards “RS” or “OS” can inhibit VEGF angiogenic response [39].

There is growing evidence that glutathionyl hemoglobin may be of use as a biomarker of OS in circulation [40, 41]. GSH also functions in the detoxification of xenobiotics, which are eventually converted to the mercapturic acids and excreted through urine or feces [42].

GSH can also achieve a prooxidant effect but to a lesser extent than antioxidant effect [21]. During the GSH catabolism, removal of the *γ*-glutamate residue from the cysteine residue caused a prooxidant effect and may induce lipid peroxidation of the plasma membrane on the exposed, outer side [43, 44]. This may cause initiation of a signaling process inside the cell and increased production of reactive species and further cause DNA damages and lipid peroxidation [43, 45]. Moreover, the prooxidant effect of GSH can enhance the reduction of iron and oxidation of low-density lipoproteins (LDL) involved in vascular injury and atherogenesis development [21].

## 3. Glutathione and Reductive Stress

Albert Wendel coined reductive stress, to describe NADH facilitating a reduction of chelated ferric iron when excessive concentrations of NADH are present [46]. It is now known that RS is the counterpart of OS that is characterized by excessive levels of reducing bioequivalents [47]. The endogenous intracellular antioxidant, GSH, was shown to be involved in several RS-related mechanisms.

Salvemini et al. [48] showed that the HeLa cells when transfected with the human glucose-6-phosphate dehydrogenase (G6PD) gene, responsible for the generation of NADPH, exhibited increased levels of GSH and decreased ROS production. Moreover, these clones displayed significant resistance to oxidant-mediated cell killing and resistance to NF-kappaB activation [48]. Thus, these clones represent a reduced state to a certain extent.

Heat shock proteins (HSPs) were also shown to exhibit protection against several stress stimuli in mammalian cells. In line with this fact, Preville et al. [49] demonstrated that human heat shock protein 27 (Hsp27)—and murine (L929 fibroblast) heat shock protein 25 (Hsp25)—mediates protection against H_2_O_2_-induced OS by increasing levels of reduced GSH in a G6PD-dependent manner. Also, Baek et al. [50] demonstrated that the overexpression of Hsp25 enhances radiation survival in L929 cells by reducing apoptosis. However, these clones also showed increased concentrations of GSH, not as a consequence of glutathione synthesis but rather a consequence of GSSG being reduced faster to GSH. Thus, the GSH/GSSG ratio was significantly less in the controls when compared with the clones. These reports provide the first evidence that HSPs help facilitate the glutathione-redox cycle by increasing GSH levels thereby promoting a reduced state [50].

McMahon et al. [51] demonstrated that Kelch-like ECH-associated protein 1 (Keap1)-dependent proteasomal degradation of regulatory protein Nrf2 contributes to the decreased expression of several antioxidant enzymes. It has been shown in a study by Zhang et al. [52] that cardiac-related adaptation to chronic stress is facilitated by NADPH oxidase-4 (NOX4). Brewer et al. [53] showed the connection between these studies by demonstrating that NOX4 activated Nrf2 which facilitates the expression of antioxidant-related genes, which resulted in increased concentrations of GSH and consequently an increased GSH/GSSG ratio.

Rajasekaran et al. later demonstrated that increased levels of GSH, NADPH, and antioxidative pathway enzymes associated with RS, and decreased OS biomarkers could be linked to protein aggregation cardiomyopathy and cardiac hypertrophy [54]. Activation of reactive persulfides and polysulfides that have better scavenging activity than GSH can also cause “RS-related redox collapse,” but this is not well studied [55]. Nonetheless, these shifts towards reduction that induced the “RS-related redox collapse” have been linked to several complications including lipid damage [56], cytotoxicity [57], mitochondrial dysfunction [57], triacylglycerol deposition [58], and cardiac ischemic injury [59].

The role of OS in the cardiovascular system (CVS) has been well demonstrated in numerous animal and human studies discussed below. However, more recent work focuses on the role of RS in CVS, as a consequence of antioxidant-based treatments often being ineffective.

In line with this thought pattern, Zhang et al. [60] explored whether overexpression of Hsp27 induces RS that results in cardiomyopathy using low to high expression levels of Hsp27 in transgenic mice. High Hsp27-expressing transgenic mice developed cardiomyopathy. Moreover, an increased GSH/GSSG ratio increased levels of glutathione peroxidase 1 (GPx-1), and decreased levels of ROS indicated that the myopathic hearts were under RS. Zhang et al. [60] then confirmed the role of RS in cardiomyopathy by demonstrating that the development of cardiomyopathy is significantly attenuated through the inhibition of GPx-1.

The link of the NADPH oxidase (NOX) protein family has been suggested for several pathologies because it produces ROS, whose excessive production leads to OS. Thus, Yu et al. [59] explored the role of NOX4 in cardiac ischemic injury using mice with cardiac-specific overexpression (CSO) of NOX4 or dominant negative NOX4. CSO of NOX4 led to OS, while the dominant negative NOX4 exhibited an increased GSH/GSSG ratio and decreased NAD(P)(+)/NAD(P)H reflective of RS. Moreover, increasing ROS production during ischemia did not recover heart function, whereas limiting levels of reducing equivalent protected the dominant negative NOX4 hearts from ischemic injury.

Here, a question arises as to whether the RS counterpart OS could alleviate such complications, in the context of redox homeostasis distorted towards the RS side. In this regard, Schulz et al. [61] demonstrated that the glucose restriction induces catalase activity and ROS formation and increases OS resistance and survival rates in *Caenorhabditis elegans*. In 2011, Ristow and Schmeisser [62] published a review focused on several such longevity-promoting interventions that are thought to converge by causing activation of mitochondrial oxygen consumption which increases ROS formation.

## 4. Glutathione Synthesis or Recuperation Deregulation

Generally, total cellular GSH content and the GSH/GSSG ratio are controlled by a GSH-negative feedback loop, as the cells undergo fluctuating OS levels. However, the general regulation of glutathione synthesis or recuperation will determine the average around which total GSH and GSH/GSSG ratio shift. This, among others, will depend on microRNAs, long noncoding RNAs (lncRNA), and mutations affecting the core synthesis or recuperation genes and their transcription (co)-factors. In light of this review, any of these factors that increase the average GSH content is of interest. As most research is focused on the detrimental effects of synthesis deficiency, there is not a lot of explicit emphasis on changes that increase the GSH content.

The miRNA miR-96-5p was shown to be upregulated in hypertrophic cardiomyopathy disease patients compared to the normal control group [63]. Kinoshita et al. [64] demonstrated that miR-96-5p increases the levels of GSH and excitatory amino acid carrier 1 (EAAC1, official name SLC1A1), the latter being a glutamate and cysteine transporter expressed on mature CNS neurons, contributing in this way to glutathione synthesis. Their results also showed the correlation of the diurnal miR-96-5p rhythm on the levels of neuronal GSH. Consequentially, the average level of miR-96-5p could be a factor determining the average level of GSH [65]. Moreover, to identify the miRNA signature for myocardial RS, Quiles et al. used the Nrf2 expression to generate mouse models exhibiting myocardial RS. These models exhibited increased GSH levels. It was demonstrated that miR-1983, miR-582-5p, miR-208b-3p, miR-1927, miR-361-5p, miR-671-5p, miR-491-5p, miR-34c-3p, and miR-96-5p were significantly upregulated. The miRNA expression profile seems to be conserved, as sulforaphane-induced RS in HL-1 cardiomyocytes exhibited increased levels of miR-208b-3p, miR-1927, miR-671-5p, and miR-96-5p as well [66].

Glutamate cysteine ligase (GCL) required for GSH biosynthesis is a heterodimer made up of glutamate-cysteine ligase modifier subunit (GCLM) and glutamate-cysteine ligase catalytic subunit (GCLC) [67]. In ovarian cancer cell lines that have high expression of GCLC, the expression of GCLC is predicted to be positively regulated by hsa-miR-133a and negatively by hsa-miR-140-3p. In this cellular context, GCLC is shown to exert antiapoptotic effects [68–70]. However, GCLC is also known to be tightly regulated with the negative feedback loop through the Nrf2-Keap1 interplay. There is evidence that Nrf2 expression is directly regulated by miR153, miR27a, miR142-5p, and miR144 independently from the Keap1 interaction [71, 72]. Keap1 mRNA can be targeted by miR-200a, lowering its expression and releasing more Nrf2 into the nucleus, whereas Nrf2 nuclear export is regulated by sirtuin1 (Sirt1) which is targeted by miR-34a [73]. GCLC is also regulated by LINC00942; this lncRNA can be targeted with antisense oligonucleotides to reduce expression of GCLC. It seems to be involved purely with the transcription of GCLC, and LINC00942 is itself transcriptionally regulated by Nrf2 [74]. Clinically relevant variants have been reported for GCLC that affect its binding with GCLM. GCLC protein of Cys248Ala, Cys249Ala, and Pro158Leu variants shows similar binding strength with GCLM as did wild-type GCLC, but they have a large decrease in catalytic activity. Higher quantities of GCLM can sometimes counterbalance the impact of these less efficiently binding variants [75, 76]. For GCLM, an upstream variant C588T has been reported. Using a luciferase reporter gene assay, the variant was shown to have lower promoter activity in response to oxidants and consequently lower GSH levels [77]. GCLC also has a relatively close intergenic LNC RP11-345L23.1 (LINC01564) at 11 kb that could be relevant for its expression [74].

Expression of circulating miR-92a, let-7c, miR-145, and miR-155 was also shown to be significantly reduced in coronary artery disease patients in comparison to the control group [78]. Overexpression of miR-145 decreases the expression of the prostate-specific androgen-regulated lncRNA, Pcgem1 [79]. Pcgem1 regulates expression of glutaminase, type I gamma-glutamyltransferase, and glutathione reductase (GSR), all involved in the metabolism of glutamine, a precursor of glutathione [80]. The overall effect of decreased Pcgem1 is the decreased production of glutathione.

**Figure 1:**
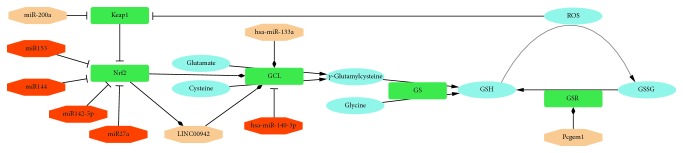
Overview of key components involved in CV-related reductive stress. Metabolites are shown in blue ovals, proteins in green rounded rectangles, and RNAs (microRNA or lncRNA) in octagonals. If the latter have a positive impact on GSH content, they are colored peach; if negative, they are orange. GSH: glutathione; GCL: glutamate cysteine ligase; GSSG: glutathione disulfide; GS: glutathione synthetase; Keap1: Kelch-like ECH-associated protein 1; Nrf2: nuclear factor erythroid 2-related factor 2; ROS: reactive oxygen species; GSR: glutathione reductase.

The combined result of microRNAs, mutations of key proteins, and to a lesser extent, lncRNA regulation will set a tissue-specific average level of GSH (Figure 1). When this is low, it can lead to the diseases caused by OS; on the other hand, a high average GSH level will cause a continued state of RS with possible implications for cardiovascular disease.

## 5. Glutathione in CVS

### 5.1. Evidence from Animal Studies

OS leads to a number of diseases, from neurodegenerative disease [81] to CVD and diabetes type II [82]. Using the model of atherosclerosis in mice or the apolipoprotein E-deficient mice, Rosenblat et al. [83] reported that liposomal coated GSH in a dose of 50 mg/kg/day for two months showed significant reduction in serum susceptibility of 2,2′-azobis(2-amidinopropane) dihydrochloride (AAPH) oxidation, in a correlation with an increased level of GSH content in peritoneal macrophages, reduction in lipid peroxides [83], and also a decrease of the extent of oxidized LDL. Another study by Lin et al. showed that N-acetyl cysteine (NAC) could increase GSH levels, and consequently, GSH can reduce significantly cholesterol levels in the liver and plasma in mice that are on a high saturated fat diet [84]. One explanation is that GSH increases the expression of cholesterol 7 alpha-hydroxylase, thus resulting in the increased biosynthesis of bile acids from cholesterol [84, 85]. Moreover, depressed GSH synthesis will lead to and/or precede OS and atherogenesis [86]. These results present a novel pathway of how glutathione in this form has antioxidative and antiatherogenic properties and may lead to remission of atherosclerosis [83, 87].

In the hyperglycemic state [88], the production of nitric oxide (NO) and a reduction of antioxidant effects are increased. Using alloxan-induced hyperglycemia into rabbits, the authors investigated the level of nitrotyrosine (as a stable product of nitrosative stress) as a marker for NO-dependent damages. Also, in the same study, the level of GSH was measured. Hyperglycemic rats show that tissues differ (heart vs. brain, liver) in neutralizing nitrosative stress, and this process is placed by different adaptive responses of their GSH redox cycle [88].

In a previous study [89] using the same experimental model, the authors analyzed the effect of pioglitazone, an antidiabetic drug, on the heart. GSH, GSR, GPx, lipid peroxidation products, ascorbic acid, and other parameters were measured. The result from this study shows that pioglitazone increased the GSH system parameters, thus reducing OS, and the authors proposed that similar action improves atherosclerotic problems in diabetic patients.

In the process of atherogenesis, GSH capacity to be synthesized in cells, but especially in macrophages, has an inverse relationship to the initiation and progression of atherosclerosis in ApoE^−/−^ mice [90]. Results from animal models (hyperlipidemic mice) show that a crucial event for the progression of early vascular lesions (atherosclerotic plaque) is GSH plasma concentrations [83, 90]. These results are supported by the results showing that bone marrow transplants are capable of synthesizing 3x more GSH than normal and show the reduced progression of lesions up to 35% [90]. Reduction in Lp(a), apoB, LDL-c, and total plasma cholesterol has been reported in transhumanized mice with proatherogenic lipid metabolism after administration of ribose cysteine that increases the level of GSH and GPx activity [91].

Glutathione peroxidase 1 is implicated in DM-associated atherogenesis [92]. GPx catalyzes the reaction between glutathione and hydrogen peroxide and is the most abundant (type 1) in all mammalian tissues. In a diabetic apolipoprotein E-deficient mouse model [92], decreased levels or lack of GPx-1 accelerates diabetes-associated atherosclerosis. Other animal studies [93, 94] showed that GPx-1 when reduced increases the level of the cell LDL oxidation process. These results show that GPx-1 which is present in the mitochondria and the cytosol is a critical enzyme in the protection of vessels from OS and atherogenesis.

Altered silencing of protein activity by noncoding RNA fragments (miRNA) has been proposed to be a mechanism that may impact CVD [95]. New research [96] shows that microRNAs are possible regulators of expression of GPx in obesity-related pathologies. miRNA 494 was found in an *in vivo* model to regulate plaque size and the stability of the lesions and decrease the occurrence of rupture [97]. miRNA 27a and miRNA 223 contribute to cholesterol homeostasis [98, 99]. Also, Milenkovic et al. [100] reported that plant polyphenols could modulate the expression miRNAs in ApoE-deficient mice.

Special attention should be made on exogenous miRNA as it can be absorbed by our diet and by the gastrointestinal tract and reaching plasma levels in stable microvesicles [101] and consequently modulate and influence a number of antioxidant proteins, including GSH [102].

Results from *in vivo* studies show that pharmacological interventions modestly protect against the development of early fat streak in the aortic sinus [87]. These results coincide with human intervention studies that show that antioxidant supplementation does not coincide with any progress in the attenuation of CVD in mice and humans [103, 104]. Furthermore, interventions on the GSH system by increasing its endogenous levels show a promising strategy to enhance its antiatherogenic effects [105–108]. Changes in the GSH content in macrophages also affect NF*κ*B and other proinflammatory cytokines that are responsible for the stimulation adhesion molecules in endothelial cells and recruitment of monocytes or macrophages in arterial lesions [109, 110]. These effects are strengthened by the notion that increasing and decreasing levels of antioxidants of the GSH system in macrophages are sufficient to impact the already established atherosclerotic process [108].

### 5.2. Evidence from Human Studies

There are numerous results from both animal (Table 1) and human (Table 2) studies showing beneficial effects of GSH on CVS [111, 112]. Depletion of GSH increases predisposition to OS and leads to the occurrence of many diseases, including CVD. Several studies reported that patients with heart disease and diabetes have a lower level of plasma GSH [113, 114].

One of the first retrospective population-based case-control studies that evaluated the level of GSH in patients with CVD was conducted in the mid-90s in Japan. In the Hisayama study, Shimizu et al. [113] examined the level of total GSH in plasma and red blood cells of 134 patients with stroke and myocardial infarction. Results show that the increased level of GSH leads to decreased values of systolic and diastolic pressures and that the increased level of GSH is also followed by a decreased incidence of diabetes. Furthermore, the same authors reported that patients with CVD have a lower level of GSH compared with control subjects with no previous history of CVD [113]. Similar results were obtained in another study, where decreased levels of GSH and enzymes involved in GSH synthesis were measured in patients with type 2 diabetes mellitus (T2DM). In contrary, the level of GSSG and transforming growth factor beta (TGF-*β*) was significantly higher in diabetic patients compared with healthy controls. In this study, the authors demonstrated that the increased level of proinflammatory cytokines (e.g., TGF-*β*) decreases expression of enzymes involved in GSH synthesis, such as GS and GCL, and thus affect GSH decrease faster than it could be synthesized [115].

Chaves et al. [116] in order to assess the role of OS in hypertension measured the levels of GSH and GSSG in mononuclear cells of 38 control subjects and 35 patients with nontreated hypertension. Parameters of OS were measured in both groups, at the beginning of a study and three months after the administration of different antihypertensive therapies to individuals with hypertension. Results show that the level of GSH was decreased in hypertensive patients while the level of GSSG was increased compared with control subjects. On the other hand, three months of antihypertensive therapy decreased the level of OS and GSSG and increased the level of GSH in hypertensive patients [116]. Similar results were obtained in an observational study by Redon et al. [117] where the level of GSH was decreased while the level of GSSG was increased in mononuclear cells of 66 hypertensive patients compared with 16 control subjects without hypertension. Authors pointed out the importance of OS in the onset of hypertension and further development of the CVD [116, 117]. Also, Robaczewska et al. [118] suggested that the level of GSH and enzymes involved in the synthesis of GSH is disturbed in old people with diagnosed hypertension. Rybka et al. [119] went a step further and investigated the effect of different types of antihypertensive drugs on the level of GSH and enzymes involved in the synthesis of GSH in old people. The study was conducted on 18 patients diagnosed as hypertensive who were on antihypertensive therapy and 15 healthy controls. Measurement of the level of GSH, the activity of GSH, and other values of all antioxidant enzymes was higher in hypertensive patients on antihypertensive therapy compared with healthy subjects, and this hint that antihypertensive therapy has positive effects on the antioxidant system in elderly people [119].

Damy et al. [120] evaluated results of the level of GSH in 76 patients who had some form of cardiac surgery (heart transplantation, coronary artery bypass grafting, ventricular assist device implantation, and aortic valve replacement). In this study, the level of GSH was measured during surgery in right atrial appendages and blood. The authors noticed that the lower level of GSH was in patients with coronary artery disease and that this decrease in the GSH level was consistent with the severity of left ventricular dysfunction [120]. Additionally, in comparison with healthy controls, the level of GSH was 21% and 40% decreased in patients with asymptomatic and symptomatic CVD, respectively. From these results, the authors concluded that decreases in the level of GSH are closely linked to cardiac abnormalities in patients with CVD. Furthermore, since these results show that the level of GSH was also decreased in patients with still undetected CVD, authors suggested that a blood test for measuring the level of GSH should be used as a new biomarker for detection of asymptomatic patients with CVD [120].

During cardiac procedures, increased OS could lead to myocardial infarction (MI). Glutathione S-transferase (GST) polymorphism is identified as one of the factors that could lead to an increased incidence of MI during cardiac surgery. To investigate the association between GST polymorphism and MI, Kovacs et al. [121] conducted a study on 758 patients that had cardiac surgery. After measuring levels of troponin 1 (T1) and myocardial-based creatine kinase (CKMB), two groups of patients were formed. The control group consisted of 78 patients, with no signs of MI and with double values of T1 and CKMB after surgery, while the second group consisted of 54 patients, with signs of MI after cardiac surgery and with five times higher values of T1 and CKMB. Both groups of patients underwent genetic testing for the presence of GST polymorphism (GST P1, alleles A, B, and C). Results show that the presence of BB allele was higher in the control group of the patient without MI. On the other hand, allele AC was detected in a group of patients with MI. Authors suggested that the presence of allele B may have a protective role in the development of MI, while the presence of alleles A and C was associated with increased risk for MI [121].

Glutathione peroxidase has an important role in OS. Decreased activity of GPx-1 increases risks for stroke and coronary heart disease [122, 123]. The level of erythrocyte GPx-1, in a study of 83 patients who died from some form of CVD or had a myocardial infarction and 553 control subjects without any CVD, was lower in patients with CVD compared with control subjects [123]. These results show that GPx-1 is inversely associated with CVD and also that GPx-1 is important for maintenance of a normal level of GSH. Authors predicted that measuring the level of erythrocyte GPx-1 could be used as prognostic value and that increasing the level of GPx-1 could have a beneficial effect on CVS [123].

Investigation of connection between GPx-1 polymorphism and development of atherosclerosis in 184 Japanese patients with the T2DM show that GPx-1 is the most important enzyme, with the protective role in the development of endothelial dysfunction and atherosclerosis in diabetes. In this study, patients were divided into two groups, depending on the presence of GPx-1 genotype (Pro/Pro: *n* = 151; Pro/Leu: *n* = 33), and intima-media thickness (IMT) of carotid arteries was measured. Results show higher values of IMT in the Pro/Leu group compared with values of IMT measured in the Pro/Pro group of subjects. Since increased IMT values are positively related with the onset of atherosclerosis, authors concluded that incidence of CVD was higher in a group of patients with GPx-1 Pro/Leu genotype, which is also consistent with obtained values of IMT measurement [94].

Gene expression for biosynthesis of glutamate-ammonia ligase depends on single nucleotide polymorphism (SNP) rs10911021. SNP rs10911021 is also associated with coronary heart disease (CHD) in diabetic patients. In a study with 425 patients with CHD, where 275 of them were diabetic, a direct association between SNP rs10911021 and diabetes was found among CHD patients. The level of GSH was lower, while the GSSG level was higher in patients compared with controls. These results suggest that the presence of SNP rs10911021 may affect the risk for an increase of CHD in diabetes by promoting OS [124].

De Mattia et al. [125] in a randomized, double-blind cross-over study with 15 diabetic patients tested the hypothesis that the level of vascular cell adhesion protein 1 (VCAM-1), which is increased in atherosclerosis, could be decreased by administration of antioxidant agents. Patients received oral NAC in a dose of 1200 mg per day or placebo for one month, and the results show that administration of NAC increased levels of GSH as well as the ratio GSH : GSSG, while reduced levels of VCAM-1 and GSSG. The decrease of endothelial adhesion molecules after NAC treatment could prevent vascular damage in patients with diabetes [125].

In an open-label pilot study, Szkudlinska et al. [126] tested their hypothesis that oral administration of NAC decreases markers of OS, increases levels of GSH, and thus improves *β*-cell function in patients with diabetes. For 30 days, 13 subjects with T2DM were on NAC treatment. During the first two weeks, subjects were treated with oral NAC in a dose 600 mg, twice a day, and for the last two weeks twice a day with a double dose of oral NAC (1200 mg). Markers of OS, GSH, and GSH/GSSG were measured after two and four weeks of NAC supplementation. At the end of study, authors concluded that oral administration of NAC had no effect in patients with T2DM and that levels of GSH and GSH/GSSG remain unchanged [126].

Most of the studies where attention was on the effects of oral administration of GSH show that the level of GSH remains the same in cells, especially in red blood cells [127]. Effect of oral GSH on markers of OS (GSH, GSSG, and GSH : GSSG) was tested in 40 healthy adults. After four weeks of oral administration of GSH (500 mg twice a day), no change in markers of OS was observed [128]. In contrary, in a 6-month placebo-controlled, randomized, double-blinded trial effects of different doses (low dose of GSH (250 mg/day) or high dose of GSH (1000 mg/day)) of oral administration of GSH on the level of GSH in lymphocytes, erythrocytes, and plasma show that the level of GSH was increased in lymphocytes, erythrocytes, and plasma in the high-dose group of patients, while in the low-dose group of patients, increases were detected in erythrocyte counts only. Furthermore, results from the same study show that the increased level of GSH was only observed during administration of oral GSH supplementation, and after one month without treatment, the level of GSH returned to normal [111].

Oral administration of GSH may not be the best solution since it was shown that intestinal and hepatic GGT have the ability to metabolize GSH and thus decrease the level of administered GSH [129]. To evaluate the level of GSH in blood, Buonocore et al. [130] analyzed effects of pure GSH in the form of an orobuccal tablet with a fast-slow release on 15 healthy volunteers and concluded that the increased level of GSH in blood is probably a result of GSH absorption through mouth mucosa. In a randomized crossover study performed by Schmitt et al. [129], authors compared the level of GSH and other markers of OS in the blood of 20 subjects with metabolic syndrome after administration of different forms of GSH (oral and sublingual) and NAC. For three weeks, randomly selected subjects received oral or sublingual GSH in a 450 mg dose or a NAC in a 200 mg dose. The experiment was repeated two more times, with two weeks without treatment before the next administration of GSH or NAC. Results show that administration of sublingual GSH compared to oral GSH leads to an increase in the level of GSH and the GSH/GSSG ratio. Also, increased levels of GSH and GSH/GSSG were detected comparing the effects of sublingual GSH with NAC. Since overproduction of ROS is involved in the development of metabolic syndrome, authors concluded that administration of the sublingual form of GSH could be a possible treatment for decreasing OS and preventing the occurrence of metabolic syndrome [129]. Another study on human subjects investigated the effect of intracoronary infusion of GSH to patients admitted to the hospital for chest pain and which were planned for cardiac catheterization. All 26 patients were injected with acetylcholine (Ach) (50 mg/min) into the left coronary artery. After 15 minutes, 14 patients received in the same manner GSH (50 mg/min for 6 min), while the remaining 12 patients were treated with the same dose of saline. Authors noticed that a combination of Ach and GSH has vasodilatory effects on coronary arteries and increases blood flow but does not affect blood pressure. On the other hand, no effect was noticed in patients that received a combination of Ach and saline. Authors concluded that GSH has positive effects on CVS, increases dilatation of human arteries, and suggest that these positive effects could be mediated via activation of NO synthase or guanylate cyclase [112].

## 6. Concluding Remarks

Glutathione plays an important etiological role in the development of numerous diseases, such as cardiometabolic disease and CVD [6–9]. To avoid negative health consequences, the redox homeostasis has to be preserved, with glutathione as one of the key etiological factors in these processes. Despite many available literature data, the role of glutathione, in both normal and pathological conditions, such as CVD, still remains unclear. The literature data discussed in this review that are related to the effects of glutathione, the most abundant antioxidant in the heart, in CVS, suggests that glutathione has an important role in cell redox homeostatic mechanisms that have been shifted towards OS or RS. Further studies should focus on the understanding of the molecular mechanisms underlying the effects of glutathione in physiological conditions as well as in pathological conditions.

## Figures and Tables

**Table 1:** Evidence from animal studies.

Model	Species	Treatment	Effects	Ref
*ApoE* ^−/−^	Mice	Liposomal coated GSH 50 mg/kg/day for 2 months	Reduction of AAPH oxidation and lipid peroxides and oxidation of LDL	[83]
*ApoE* ^−/−^	Mice	OTC 500 mg/kg/day for 6 weeks	Increased level of GSH, reduction of cellular OS, and oxidation of LDL	[87]
*ApoE* ^−/−^	Mice		GSH is depleted in the atheroma-prone aortic arch	[86]
*ApoE* ^−/−^/*GCLM*^−/−^	Mice		Reduced level of GSH; atherogenesis	[90]
High saturated fat diet	Mice	N-Acetyl cysteine, oral for 4 weeks	Increased level of GSH; reduced cholesterol level in plasma and the liver	[84]
Transgenic human lipoprotein(a)	Mice	Ribose-cysteine 0.16 g/kg/day for 8 weeks	Increased level of GSH and GPx activity; antiatherogenic effect	[91]
Endothelial cells	Rat	Pretreatment with H_2_O_2_ 24 h and Zn supplementation	Increased expression of GCS and synthesis of GSH	[106]
Macrophage cells	Murine	Homocysteine 50 *μ*M	Reduced level of GSH; increased OS and GCS activity	[105]
Alloxan-induced hyperglycemia	Rabbit		Different GSH redox cycles in different tissues (heart, brain, and liver)	[88]
Alloxan-induced hyperglycemia	Rabbit	Pioglitazone	Increased GSH system parameters	[89]

AAPH: 2,2-azobis(2-amidinopropane) dihydrochloride; *ApoE*^−/−^: apolipoprotein E-deficient mice; *ApoE*^−/−^/*GCLM*^−/−^: mice doubly deficient in apolipoprotein E and *γ*-glutamylcysteine synthetase; BSO: buthionine sulfoximine; DEM: diethyl maleate; GCS: *γ*-glutamyl cysteine synthetase; GSH: glutathione; GPx: glutathione peroxidase; H_2_O_2_: hydrogen peroxide; OS: oxidative stress; OTC: L-2-oxo-4-thiazolidin carboxylate (which supplies cysteine residues); Zn: zinc (in form ZnSO_4_).

**Table 2:** Evidence from human studies.

Group	Condition	Treatment	Effects	Ref
CVD	Stroke/MI		Decreased level of GSH	[113]
CVD	MI after cardiac surgery		GST polymorphism; presence of allele AC	[121]
CVD	MI/death from some form of CVD		Decreased level of erythrocyte GPx-1	[123]
CVD	Heart transplantation/coronary artery bypass grafting/ aortic valve replacement		Decreased level of GSH	[120]
CVD	Cardiac catheterization	Ach (50 mg/min) with GSH (50 mg/min) or saline (50 mg/min)	Vasodilatory effects on coronary arteries and increased blood flow	[112]
Diabetes/CVD	Type 2 diabetes mellitus/some form of CVD		Increased values of IMT in Pro/Leu GPx-1 genotype	[94]
Diabetes/CVD	Diabetes/coronary heart disease		Decreased level of GSH; increased level of GSSG	[124]
Diabetes	Type 2 diabetes mellitus		Decreased levels of GSH and enzymes involved in GSH synthesis; increased level of GSSG and TGF-*β*	[115]
Diabetes	Type 2 diabetes mellitus	NAC in a dose 1200 mg/day for 1 month	Increased levels of GSH and GSH : GSSG ratio; decreased levels of VCAM-1 and GSSG	[125]
Diabetes	Type 2 diabetes mellitus	Oral NAC (600 mg/2x daily/2 weeks) and oral NAC (1200 mg/2x daily/2 weeks)	Unchanged levels of GSH and GSH/GSSG ratio	[126]
Hypertension	Elderly people with hypertension		Disturbed level of GSH and enzymes involved in the synthesis of GSH	[118]
Hypertension	Elderly people with hypertension	Antihypertensive drugs	Increased level of GSH and GSR	[119]
Hypertension	Hypertension		Decreased level of GSH; increased level of GSSG	[117]
Hypertension	Nontreated hypertension	Different antihypertensive therapies for 3 months	Decreased level of OS and GSSG; increased level of GSH	[116]
Metabolic disorder	Metabolic syndrome	3 weeks with oral NAC (200 mg/day), oral GSH (450 mg/day), or sublingual GSH (450 mg/day)	Increased levels of GSH and GSH/GSSG ratio in sublingual GSH	[129]
Control	Healthy adults	Oral GSH in a dose 500 mg twice a day for 4 weeks	Unchanged markers of OS	[128]
Control	Healthy adults	Oral GSH; low dose (250 mg/day for 6 months) or high dose (1000 mg/day for 6 months)	High-dose group: increased level of GSH in lymphocytes, erythrocytes, and plasma; low-dose group: increased level of GSH in erythrocytes	[111]
Control	Healthy adults	Orobuccal GSH	Increased level of GSH	[130]

GSH: glutathione; NAC: N-acetyl cysteine; OS: oxidative stress; VCAM-1: vascular cell adhesion protein 1; GSSG: glutathione disulfide; IMT: intima-media thickness; GPx: glutathione peroxidase; TGF-*β*: transforming growth factor beta; MI: myocardial infarction; Ach: acetylcholine.

## Abbreviations

Ach: Acetylcholine
CVS: Cardiovascular system
CVD: Cardiovascular disease
CHD: Coronary heart disease
CSO: Cardiac-specific overexpression
CKMB: Creatine kinase myocardial-based
GSH: Glutathione
GCL: Glutamate cysteine ligase
GSSG: Glutathione disulfide
GS: Glutathione synthetase
GST: Glutathione S-transferase
GPx: Glutathione peroxidase
GSR: Glutathione reductase
GGT: Gamma-glutamyl transferase
GCLM: Glutamate-cysteine ligase modifier subunit
GCLC: Glutamate-cysteine ligase catalytic subunit
G6PD: Glucose-6-phosphate dehydrogenase
Hsp27: Heat shock protein 27
IMT: Intima-media thickness
Keap1: Kelch-like ECH-associated protein 1
LMW-PTP: Low molecular weight protein tyrosine phosphatase
LDL: Low-density lipoproteins
lncRNA: Long noncoding RNA
MI: Myocardial infarction
NAC: N-Acetyl cysteine
Nrf2: Nuclear factor erythroid 2-related factor 2
NOX4: NADPH oxidase-4
OS: Oxidative stress
ROS: Reactive oxygen species
RS: Reductive stress
T2DM: Type 2 diabetes mellitus
TGF-*β*: Transforming growth factor beta
T1: Troponin 1
VCAM-1: Vascular cell adhesion protein 1
VEGF: Vascular endothelial growth factor.
